# The Ambiguous Role of Caffeine in Migraine Headache: From Trigger to Treatment

**DOI:** 10.3390/nu12082259

**Published:** 2020-07-28

**Authors:** Magdalena Nowaczewska, Michał Wiciński, Wojciech Kaźmierczak

**Affiliations:** 1 Department of Otolaryngology, Head and Neck Surgery, and Laryngological Oncology, Faculty of Medicine, Ludwik Rydygier Collegium Medicum in Bydgoszcz, Nicolaus Copernicus University, M. Curie 9, 85-090 Bydgoszcz, Poland; 2 Department of Pharmacology and Therapeutics, Faculty of Medicine, Collegium Medicum in Bydgoszcz, Nicolaus Copernicus University, M. Curie 9, 85-090 Bydgoszcz, Poland; wicinski4@wp.pl; 3 Department of Sensory Organs Examination, Faculty of Health Sciences, Collegium Medicum in Bydgoszcz, Nicolaus Copernicus University, M. Curie 9, 85-090 Bydgoszcz, Poland; wojciech.kazmierczak@umk.pl

**Keywords:** migraine, headache, caffeine, coffee, trigger, withdrawal headache, adenosine, vasoconstriction, cerebral blood flow

## Abstract

Migraine is a chronic disorder, and caffeine has been linked with migraine for many years, on the one hand as a trigger, and on the other hand as a cure. As most of the population, including migraineurs, consume a considerable amount of caffeine daily, a question arises as to whether it influences their headaches. Indeed, drinking coffee before a migraine attack may not be a real headache trigger, but a consequence of premonitory symptoms, including yawning, diminished energy levels, and sleepiness that may herald a headache. Here, we aim to summarize the available evidence on the relationship between caffeine and migraines. Articles concerning this topic published up to June 2020 were retrieved by searching clinical databases, and all types of studies were included. We identified 21 studies investigating the prevalence of caffeine/caffeine withdrawal as a migraine trigger and 7 studies evaluating caffeine in acute migraine treatment. Among them, in 17 studies, caffeine/caffeine withdrawal was found to be a migraine trigger in a small percentage of participants (ranging from 2% to 30%), while all treatment studies found caffeine to be safe and effective in acute migraine treatment, mostly in combination with other analgesics. Overall, based on our review of the current literature, there is insufficient evidence to recommend caffeine cessation to all migraine patients, but it should be highlighted that caffeine overuse may lead to migraine chronification, and sudden caffeine withdrawal may trigger migraine attacks. Migraine sufferers should be aware of the amount of caffeine they consume and not exceed 200 mg daily. If they wish to continue drinking caffeinated beverages, they should keep their daily intake as consistent as possible to avoid withdrawal headache.

## 1. Introduction

Migraine has emerged as a great public health concern, and the World Health Organization (WHO) has classified it as the third most common disease worldwide, with over a billion people estimated to suffer from it [1,2]. This type of primary headache usually presents with recurrent, typically unilateral and pulsating attacks of severe headaches, lasting from 4 to 72 h, with accompanying symptoms including photophobia, phonophobia, nausea, and vomiting [3]. Caffeine has been linked with migraine for many years, on the one hand as a trigger, and on the other as a cure [4,5,6,7,8]. As most of the population, including migraine sufferers, consume a considerable amount of coffee and other caffeinated drinks and foods daily, a question arises as to whether it influences their headaches. Besides, some migraine sufferers ask their doctors about dietary recommendations regarding their intake of caffeinated beverages. They demand specific information regarding whether they are allowed to drink coffee or should avoid it, and whether it will be beneficial for their migraine if they stop drinking it. The aim of this review is to examine the relationship between caffeine and migraine, and to check whether caffeine is a migraine trigger and avoiding it may be of benefit to certain patients, and to find out if caffeine may be helpful in migraine treatment. 

### 1.1. Caffeine

Caffeine is the most popular and widely used active food ingredient, with up to 80% of the population consuming a caffeinated product every day [9]. One of the most popular caffeine drinks is coffee, and many people start their day with a cup of coffee. Caffeine also occurs in tea leaves, guarana, cocoa, chocolate, cola nuts, and wide variety of medications, dietary supplements, soft drinks, and energy drinks [10]. As the structure of caffeine is similar to adenosine, it works through nonselective antagonism of adenosine A1 and A2A receptors, causing their inhibition. It is important to note that adenosine is an inhibitor of neuronal activity in the nervous system; its receptors have been reported to be involved in antinociception, and enhancing them may lead to arousal, concentration, and vigilance [11]. However, caffeine has no influence on dopamine release, thus has no potential for abuse [12]. In humans, after oral intake, caffeine is rapidly and completely absorbed (max t 30–120 min) and freely crosses the blood–brain barrier [10]. Although a main component of coffee is caffeine, it should be pointed out that it is a complex drink including over 1000 compounds, most of them not yet identified. Haskell-Ramsay compared the effects of regular coffee, decaffeinated coffee, and placebo on mood and cognition, and discovered that decaffeinated coffee also increased alertness when compared to placebo. Thus, the behavioral activity of coffee seems to expand beyond its caffeine content, and the use of decaffeinated coffee as a placebo may be controversial [13]. It is reported that moderate daily caffeine intake (300–400 mg, around 4–5 cups of coffee) is safe and does not raise any health concerns (except in pregnant women and children) [14]. Nevertheless, higher doses may induce anxiety, nervousness, headache, drowsiness, nausea, insomnia, tremor, tachycardia, and increased blood pressure [10]. Interestingly, there is evidence that response to caffeine consumption may be genetically determined [12]. Besides, the amount of caffeine that produces adverse effects can vary and is influenced by the person’s weight and sex, the presence of hypertension and hepatic disease, and metabolic induction and inhibition of cytochrome P-450 [15]. It is noteworthy that people who consume caffeine habitually have a lower risk of experiencing the adverse effects than those who do not frequently consume caffeine [10].

### 1.2. Caffeine’s Influence on Health

Coffee consumption is associated with a number of health benefits in men and women. In an umbrella review, Grosso et al. demonstrated that caffeine was associated with a decreased risk of cancer, diabetes, cardiovascular disease and mortality, and Parkinson′s disease but an increased risk of pregnancy loss [16]. On the other hand, coffee was linked with a rise in serum lipids and blood pressure. Overall, they concluded that coffee (moderate daily intake) can be part of a healthful diet [16]. A number of epidemiological studies confirmed a link between higher coffee consumption and better performance on cognitive tests in older adults, and an inverse relationship exists between coffee consumption and the risk of developing Parkinson′s or Alzheimer′s disease and a lower risk of stroke. Interestingly, regular coffee consumption does not affect patients with epilepsy [17]. It is reported that caffeine can enhance awareness, attention, and reaction time by stimulating wakefulness, increasing concentration, and decreasing the sensation of fatigue, but also may disturb sleep quality [14,17,18]. Moreover, caffeine in low doses (150–200 mg) can improve mood states and decreases the risk of depression and suicide [17].

### 1.3. Caffeine and Cerebral Blood Flow

The effect of caffeine on blood flow and arteries remains controversial. On the one hand, there is evidence that caffeine decreases the production of nitric oxide (NO, responsible for vasodilation) from the endothelial cells, and on the other hand, a number of studies showed increased NO production after caffeine administration [19,20]. Several studies investigated the direct effects of caffeine on endothelial function and concluded that caffeine augmented and improved endothelium-dependent but not endothelium-independent vasodilatation, suggesting that it has no effect on vascular smooth muscle function [21,22]. The reason for this ambiguous effect, called by Higashi the “coffee paradox,” may be a different action of caffeine on endothelium and smooth muscles [21]. It is known that caffeine is an adenosine receptor antagonist. Interestingly, adenosine via the adenosine A2A receptor stimulates the production of NO with further vasodilatation, but contrary to this, via the adenosine A1 receptor, adenosine decreases NO release and produces vasoconstriction. Thus, depending on caffeine binding affinity and dose, it can cause either vasoconstriction or vasodilatation and sometimes even no change in vascular function [21]. It is important to note that methylxanthines such as caffeine usually induce vasodilatation except in the central nervous system, where they raise cerebrovascular resistance (CVR) and reduce cerebral blood flow (CBF) [23]. A number of studies demonstrated that by inducing vasoconstriction, caffeine reduces CBF in healthy people, but also in pathological conditions. Vidyasagar at al. discovered a global 20% reduction in gray matter CBF with caffeine and tea but not decaffeinated tea, which indicates that only caffeine change CBF. Moreover, the effect of caffeine was regionally specific. Interestingly, none of the interventions had an effect on CVR [24]. Haanes et al. investigated the effect of adenosine A2A receptor antagonists on the vasodilation of the middle meningeal artery. They found that antagonists did not influence neurogenic vasodilation, but blocked the vasodilation produced by A2A receptor agonists, suggesting that selective A2A receptor antagonists might be useful in migraine treatment by preventing meningeal arterial dilation [25]. Another study, using vascular information extracted from the blood-oxygen-level-dependent (BOLD) signal in functional MRI (fMRI) showed that shorter time delays and smaller standard deviations were detected in scans of caffeinated areas. This means that caffeine increased blood flow velocity by vasoconstriction [26]. The spatial distribution of adenosine receptors may be one reason for the region-dependent changes in brain activity induced by caffeine, thus, the average brain metabolic rate stays unchanged. Besides, caffeine′s effects on arteries may be region-specific [27]. Blaha et al., in a transcranial Doppler (TCD) study, investigated the effects of caffeine on an already dilated cerebral circulation and found a significant decrease in CBF velocity after caffeine ingestion in a normal cerebrovascular bed as well as in peripheral vasodilatation. This means that caffeine may regulate CBF under various pathological conditions, with possible therapeutic effects in vasoparalysis [28]. Lunt et al., using two methods of cerebral blood flow measurement (transcranial Doppler and xenon clearance), demonstrated that 250 mg caffeine reduced CBF by an average of 22% in healthy volunteers as well as in patients recovering from stroke. Caffeine caused a smaller change in middle cerebral artery (MCA) blood flow velocity than in CBF, which indicates that caffeine reduces the MCA diameter [29]. Addicott et al. used perfusion magnetic resonance imaging to check the effect of caffeine on CBF in chronic users of low, moderate, and high amounts in an abstained state and the normal use (native) state. In each state, participants received either caffeine (250 mg) or placebo. It was found that in both states, caffeine reduced CBF by an average of 27%, but in the native placebo condition, users of high amounts of caffeine trended toward less CBF than those who consumed low and moderate amounts. These results suggest a limited ability of the cerebrovascular adenosine system to compensate for high amounts of daily caffeine [30]. Another TCD study examined whether controlled caffeine cessation would produce headache and changes in CBF velocity. After 24 h of caffeine abstinence, 10 individuals developed headache with an accompanying increase in CBF velocity. One hour after caffeine intake, the headache resolved and CBF velocity decreased. The study indicates a link between caffeine withdrawal, headache, and CBF [31]. Sigmon et al., in a double-blind study, demonstrated that acute caffeine abstinence increased mean, systolic, and diastolic velocity in the MCA and anterior cerebral artery (ACA) and decreased the pulsatility index in the MCA measured by TCD [32].

### 1.4. Caffeine′s Effects on Pain and Non-Migraine Headache

There is evidence that caffeine may reduce pain sensation through its effects on adenosine receptors [12]. The antinociceptive effects of caffeine may be explained by an inhibition of cyclooxygenase activity as well as adenosine receptor antagonism. Caffeine acts not only by central blocking of adenosine receptors, which affects pain signaling, but also by blocking peripheral adenosine receptors on sensory afferents [12]. It was demonstrated that a 200 mg caffeine dose can inhibit the analgesic effects of transcutaneous electrical nerve stimulation [33]. Caffeine (≥100 mg) combined with a standard dose of analgesics led to an increased proportion of individuals with a satisfactory level of pain relief [34]. Laska et al. found that, in combination with paracetamol or aspirin, caffeine reduced the amount of analgesic needed to reach the same effect by approximately 40% [35]. Other clinical effects in these patients may be linked with the promotion of the absorption of analgesics by rapid lowering of gastric pH. Nevertheless, meta-analyses of caffeine combined with ibuprofen, paracetamol, or acetylic acid found only weak adjuvant effects in patients with postoperative pain [34].

It has been proved that caffeine and caffeine-containing analgesics are effective in the treatment of several types of primary and secondary headaches. For example, it is known to terminate hypnic headache, a sleep-related headache disorder that wakes people from sleep at a consistent time [36]. Based on observational studies, the most effective acute and prophylactic treatment of this rare disease is caffeine [37]. Another type of headache that may benefit from caffeine is post-dural puncture headache (PDPH), the most common complication of lumbar puncture and spinal anesthesia. A Cochrane review published in 2015 revealed that treatment with caffeine reduced PDPH in a number of participants and decreased the need for supplementary interventions compared to placebo [38]. This effect is probably due to increased production of cerebrospinal fluid (CSF), as one study demonstrated that long-term consumption of caffeine induced ventriculomegaly, and adenosine receptor signaling can regulate the production of CSF [39]. It has been reported that caffeine withdrawal can often produce headaches. According to the International Classification of Headache Disorders (ICHD-3), a withdrawal headache is a headache experienced by individuals who frequently consume caffeine (>200 mg/d for >2 weeks) and suddenly stop. They develop a headache within 24 h after their last caffeine intake, which is relieved within 1 h by ingesting caffeine (100 mg) or resolves within 7 days after caffeine withdrawal [3]. The higher the baseline level of caffeine ingestion, the greater the likelihood of withdrawal headache. The cause of this type of headache is probably increased CBF due to vasoconstriction [31]. It is important to note that caffeine withdrawal has been described as the cause of reversible cerebral vasoconstriction syndrome in several cases of this rare sudden thunderclap headache [40,41]. Ward et al., in a double-blind placebo-controlled trial, examined whether caffeine alone has independent analgesic effects on non-migraine headaches, and found equivalent effects to acetaminophen [8]. According to Mazzoni et al., overuse of caffeine was found in 36.6% of patients with chronic cluster headaches, compared to only 6.9% of patients with episodic headaches [42]. Interestingly, patients with chronic daily headaches were more likely to overuse caffeine before the onset of the headache, compared with controls with episodic headaches. Nevertheless, no association was found regarding present caffeine consumption [43]. Medication overuse headache (MOH) is a rebound headache that usually occurs with frequent use of analgesics to relieve headaches (more than 10–15 days a month). Kluonaitis et al. revealed that caffeine-containing combination analgesics were overused among 35.8% of patients with migraines [44]. Another study showed that combination analgesics were the most frequently overused medications by MOH patients, and caffeine was a component of 89.9% of these [45]. A randomized double-blind study conducted in patients with tension-type headache revealed that treatment with ibuprofen and caffeine provided significantly greater analgesic effect than ibuprofen alone, caffeine alone, or placebo. Notably, no analgesic effect of caffeine alone (200 mg) compared with placebo was found [46].

### 1.5. Caffeine and Migraine

#### 1.5.1. Caffeine as Migraine Treatment: Potential Mechanism of Action 1

Although caffeine has been used for migraine headaches for many years, at the beginning its efficiency was linked with vascular properties. As caffeine produces cerebral vasoconstriction, it was thought that by this mechanism it may stop migraine attack. However, the role of vasodilatation in migraines is unclear, and recent findings challenge its necessity [47]. Nowadays, it is known that migraine is a neurological, not vascular, disorder, so the therapeutic effect of caffeine seems to be beyond its vascular effects. It is reported that adenosine is one of the neuromodulators that contribute to migraine pathophysiology. First of all, adenosine plasma levels increase during migraine attacks and exogenous adenosine may start migraine headaches [48]. Besides, an adenosine uptake inhibitor (dipyridamole) may increase the frequency of migraine attacks. Finally, as caffeine competitively antagonizes adenosine′s effects by binding to some of the same receptors, it may be effective in migraine treatment [36]. On the other hand, it is important to note that regular use of caffeine-containing analgesics is associated with medication-overuse headaches. It was demonstrated that migraine sufferers have gastric stasis not only during, but also outside of acute migraine attacks [49]. This reduction in gastric motility slows the absorption of acute medications and diminishes their effectiveness [50]. As caffeine increases gastric motility, this may have important clinical implications for migraine patients, and may contribute to its effectiveness when combined with analgesics [34]. Caffeine, by inhibiting phosphodiesterases and blocking adenosine receptors, can potentially alter nitric oxide (NO) production. Bruce et al. demonstrated that caffeine diminished exhaled NO, probably by adenosine receptor antagonism or by altering levels of cGMP [19]. As NO levels increase in jugular venous plasma during a migraine attack and NO synthase inhibitors are effective in migraine treatment, it is possible that caffeine as a biologically active compound may decrease the frequency of migraine attacks by inhibiting NO synthase production [51]. Recently González at al. found that regular coffee consumption may be associated with changes in some intestinal microbiota groups [52]. As there is a relationship between migraine and the gut–brain axis and probiotics were found to be beneficial in migraine treatment, this can be another mechanism by which caffeine may influence migraines [53,54].

#### 1.5.2. Caffeine as a Migraine Trigger: Potential Mechanism of Action

Trigger factors are events or exposures that increase the probability of an attack over a short period of time [55]. The 10 most frequent migraine triggers are stress; fatigue; fasting; auditory, visual, and olfactory triggers; hormonal triggers; sleep; weather; and alcohol [56]. Dietary triggers are less frequent, and include chocolate, coffee, red wine, nuts, cheeses, citrus fruits, processed meats, monosodium glutamate, and aspartame [57]. It is possible that an isolated trigger is insufficient to precipitate a migraine attack, thus, migraine sufferers usually recognize multiple dietary triggers [58]. Caffeine may act as a trigger in two possible ways: drinking coffee or other caffeinated beverages may start a migraine attack, and caffeine withdrawal is an even more frequent migraine trigger [59,60]. The prevalence of coffee as a migraine trigger in the reported literature ranges from 6.3% to 14.5% [36]. Moreover, caffeine overuse is one of the risk factors of migraine chronification, thus promoting the transformation of episodic migraine into its chronic form (when headaches persist for ≥15 days/month for >3 months) [61,62]. It is important to note that caffeine consumption was not significantly connected to medication overuse in chronic migraine patients [63]. A question arises: What is the exact mechanism by which caffeine can induce migraine headache? First, caffeine induces urinary loss of magnesium, probably by reducing its reabsorption [64]. As magnesium affects neuromuscular conduction and nerve transmission and plays a beneficial role in chronic pain conditions and migraines, caffeine, by decreasing the magnesium level, may induce headache [65]. Dehydration is one possible migraine trigger [66]. Caffeinated coffee in higher doses induces an acute diuretic effect, and subsequently may lead to dehydration [67]. Courturier et al. linked weekend migraine attacks to caffeine withdrawal. In their study, patients with high daily caffeine consumption on workdays and reduced or delayed intake on weekends (because of prolonged sleep) had an increased risk of weekend headache [68]. Thus, the observed higher frequency of migraines during weekends may be linked with caffeine withdrawal [68].

On the other hand, the methodological difficulties of investigating the influence of trigger factors on migraine are highlighted by many authors [58]. Premonitory features are defined as symptoms associated with an increased probability of aura or headache [55]. It is known that certain trigger factors can overlap with corresponding premonitory symptoms; for example, food craving in the premonitory phase may be responsible for eating chocolate or other foods, thus, they may be misinterpreted as migraine triggers [69]. It is possible that premonitory symptoms, including yawning, diminished energy levels, and sleepiness, may force migraineurs to drink coffee or caffeinated beverages, leading to the wrong conclusion that they triggered a migraine, while it was just a consequence of starting a migraine attack. On the other hand, premonitory sleepiness makes migraineurs prone to caffeine overuse, with further migraine chronification. Interestingly, according to Alstadhaug et al., the prodromal phase of migraine and caffeine withdrawal syndrome share the same or similar pathophysiological pathways [4].

A question arises as to whether caffeine may induce cortical spreading depression (CSD) in migraine aura sufferers. Yalcin at al. demonstrated that neither acute/chronic administration nor withdrawal of caffeine affected CSD susceptibility or related cortical blood flow changes in mice. Thus, they concluded that the influence of caffeine on headache is not linked with CSD pathophysiology, which may explain the non-migrainous presentation of caffeine-related headache [70].

Thus, should migraine patients strictly avoid all potential triggers, including caffeine? First of all, trigger avoidance create frustration, which may limit the beneficial effects or make the situation worse. Moreover, migraine is a disorder of the habituation of the CNS to sensory signals, thus, the brain should be trained to habituate to, not avoid triggers [71]. It is reported that short exposure to a headache trigger may increase sensitivity, while chronic exposure results in diminished sensitivity (leading to desensitization). According to Martin et al., patients with migraines should cope with triggers rather than avoid them [72].

If caffeine is a migraine trigger, does its cessation influence migraine attack frequency? Mikulec et al. demonstrated that only 14% of vestibular migraine patients reported an improvement in symptoms upon caffeine cessation [73]. Lee et al. evaluated the effect of caffeine cessation on the acute treatment of migraine. After controlling for covariates, caffeine cessation was independently connected with excellent efficacy of acute treatment. Indeed, 72.2% of those in the abstinence group reported excellent efficacy of triptans compared with only 40.3% in the non-abstinence group (*p* = 0.002). Besides, the abstinence group trended toward a greater reduction in headache impact test-6 (HIT-6) scores [74]. On the other hand, Mostofsky et al. revealed no association between one to two servings of caffeinated beverage intake and the odds of headaches on that day; only three or more servings were linked with higher odds of headache [75]. As caffeine dose per serving varies by type of drink and preparation method it may be difficult to assess the amount with increased risk. The average caffeine content of an 8 oz cup of coffee is around 100 mg [10]. It means that migraineurs may consume up to 200 mg caffeine without increased attacks risk.

**Figure 1:**
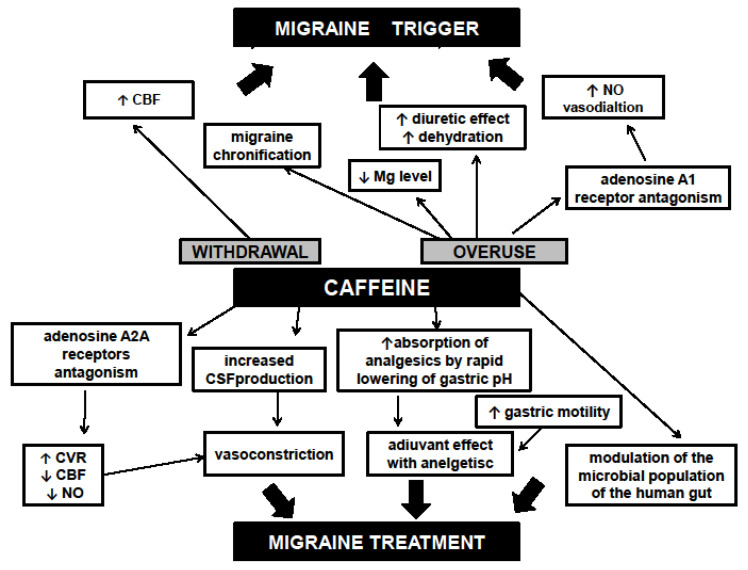
Possible mechanisms by which caffeine may trigger or stop migraine attacks (based on our literature review). Abbreviations: CBF—Cerebral blood flow, CVR—Cerebrovascular resistance, CSF—Cerebrospinal fluid, NO—Nitric oxide, Mg—Magnesium.

All possible mechanisms regarding the influence of caffeine on migraine headache are summarized in Figure 1.

## 2. Materials and Methods

This review includes all articles concerning the association between migraines and caffeine/coffee published up to June 2020. The list was obtained by searching clinical databases, including the PubMed, MEDLINE, Google Scholar, and Cochrane Library databases. Papers regarding any connection between caffeine/coffee and migraine were identified through a literature search. The applied terminology and keywords included “caffeine”, “coffee”, “caffeine withdrawal”, “adenosine”, “migraine”, “headache”, “trigger factors”, “treatment”, and “pain”. Each article was then cross-referenced to identify relevant studies. Only English language studies were eligible for inclusion. All types of articles, including clinical trials, observational, cross-sectional, and case-control studies, were involved and reviewed. Two independent investigators extracted data from each article.

## 3. Results and Discussion

### 3.1. Prevalence of Caffeine as a Migraine Trigger Factor

All studies investigating the prevalence of caffeine/coffee or caffeine withdrawal as a trigger factor in patients with migraines are summarized in Table 1.

Twenty-one studies evaluated the prevalence of caffeine as a migraine trigger. Among them, four studies failed to find any participant who reported caffeine as a trigger. In other studies, caffeine was reported to be a migraine trigger in a small percentage of participants (ranging from 2.4% to 30%). Only two studies examined caffeine withdrawal as a trigger factor, both with a relatively high percentage of patients (ranging from 10% to 30%) [60,86]. However, it is worth noting that in most of the studies, patients were asked retrospectively to recall their usual headache triggers using a predetermined list, thus they mostly assessed beliefs about triggers rather than facts. Only one study used an electronic diary (supposedly one of the best trigger factor study designs) and found coffee as a trigger in a very small percentage of migraineurs. Unfortunately, we found no provocative studies evaluating whether caffeine can provoke migraine attacks. It is reported that a high level of caffeinated beverage intake may induce a migraine attack on that day. Mostofsky et al., in a prospective cohort study, found that although consuming one or two caffeinated beverages was not associated with the odds of having a migraine on that day, ≥3 beverages was connected to higher odds of having a headache, even after accounting for potential confounding by other triggers. Moreover, a nonlinear association between caffeine intake and the odds of migraine occurrence on that day was found [75]. Taheri et al. examined the effects of dietary exclusion on the course of primary headache including migraine in a group of children. Interestingly, caffeine was reported as the most common trigger in this group (28%). After excluding one to three of the identified food triggers, 87% of patients achieved complete resolution of their headaches, meaning that the cumulative effect of food rather than a single ingestion influences headaches [78]. In the Head-HUNT study, chronic headaches were more prevalent among individuals with low caffeine intake compared to those with moderate or high intake. Besides, a significant association was found between high caffeine consumption and the prevalence of infrequent headache (OR = 1.16, 95% CI 1.09–1.23). The authors concluded that high caffeine intake may change chronic headache into episodic headache due to the analgesic properties of caffeine. Another explanation is that chronic headache sufferers tend to avoid caffeine so as to not aggravate their headaches [94]. Couturier et al. revealed that weekend headaches are linked to caffeine withdrawal. They examined 151 patients with migraine or tension-type headache (TTH) and found that 21.9% of them had weekend headaches. Weekend headache sufferers consumed significantly more caffeine daily (mean 734 mg/day) and slept longer on weekends compared with those without weekend headaches. Prolonged weekend sleep delayed the usual cup of coffee, thus produced headache [68]. Camboim Rockett et al., in a very interesting study, found that coffee withdrawal was more frequently reported as a migraine trigger than coffee intake. Besides, participants reported that coffee intake produced migraine attack occasionally, but coffee withdrawal did so frequently. Moreover, coffee withdrawal was a more prevalent trigger in migraines with aura, and coffee intake was a common trigger in migraines without aura [60].

### 3.2. Caffeine as Acute Migraine Treatment

Only one prospective study evaluated separate doses of caffeine in the treatment of acute migraine attack. Baratloo et al. compared the effectiveness of either 60 mg intravenous caffeine or 2 g intravenous magnesium sulfate in migraine attacks. Although both treatment options diminished pain scores significantly, after one hour magnesium was more effective than caffeine [95]. A number of studies examined the usefulness of caffeine in combination with other analgesics. In a double-blind randomized placebo-controlled study, a combination of acetaminophen, acetylsalicylic acid, and caffeine (130 mg) was compared with ibuprofen and placebo for treatment of acute migraine in patients with severe baseline migraine pain. The combination of drugs relieved the pain and associated symptoms of severe migraine significantly better and faster than ibuprofen (*p*  ≤ 0.05) [96]. Another randomized double-blind study compared the efficacy and tolerability of the combination of paracetamol and caffeine (130 mg) with sumatriptan (50 mg) for migraine attacks. Surprisingly, both treatments were equally effective and safe with respect to the baseline, with no differences between the two [97]. In a different double-blind randomized trial, patients treated two migraine attacks, one with almotriptan 12.5 mg and one with ergotamine plus caffeine (200 mg). Almotriptan was associated with significantly greater efficacy in treating migraine compared to the combined drug, and moreover was well tolerated and associated with greater treatment satisfaction [98]. The effectiveness of a combination analgesic containing acetaminophen, aspirin, and caffeine (65 mg) was compared with ibuprofen and placebo for migraine attacks. Although both active treatments were significantly better than placebo in relieving the pain and associated symptoms of migraine, the combination product provided superior efficacy and speed of onset compared with ibuprofen [99]. Another study compared the combination of acetaminophen, aspirin, and caffeine (130 mg) with sumatriptan (50 mg) for treatment of migraine attacks. The combination product was significantly more effective (*p* > 0.05) than sumatriptan in the early treatment of migraine [100]. A randomized double-blind study evaluated the efficacy of 100 mg diclofenac sodium softgel with or without 100 mg caffeine versus placebo during migraine attacks. Headache relief at 60 min was reported by 14% of the placebo group versus 27% of the diclofenac group and 41% of the diclofenac plus caffeine group. Diclofenac softgel plus caffeine produced statistically significant benefits when compared to placebo at 60 min, while diclofenac softgel alone did not differ significantly from placebo. Nonsignificant trends support the analgesic adjuvant benefit of caffeine when added to diclofenac softgels [101].

### 3.3. Recommendations for Migraine Patients Regarding Caffeine Use

1. Individuals with migraines must be aware of the amount of caffeine they consume daily. They should carefully identify all caffeine products consumed daily, including coffee, tea, soft drinks, energy drinks, and medications.
2. Migraine sufferers who are regular caffeine consumers and wish to continue drinking caffeinated beverages, should keep their daily caffeine intake as consistent as possible. They should also choose coffee as a preferable caffeine source because of the additional health benefit. Those who wish to cease caffeine consumption should gradually taper their intake over several weeks.
3. Daily intake of caffeine should be limited to less than 200 mg/day (about two servings of caffeinated beverage).
4. Patients should continue to consume caffeine regularly every day, preferably at a consistent time, and should not discontinue it during the weekend. They should avoid sleeping longer on weekends to prevent caffeine withdrawal headache.
5. Caffeine-containing analgesics are safe and effective in treating migraine attacks, but their consumption should be limited to two days during the week to avoid medication overuse headache.

## 4. Conclusions

Although caffeine has been connected to migraine for many years, its effect on headache is ambiguous. Caffeine or coffee consumption as well as caffeine withdrawal were found to be migraine trigger factors in a small proportion of migraine patients. However, it may be challenging to distinguish between migraine triggers and premonitory symptoms, as drinking coffee or an energy drink before an attack may be due to yawning, diminished energy levels, and sleepiness that may herald a headache. Besides, no provocative studies have been conducted to confirm that caffeine can trigger migraines. On the other hand, caffeine alone or as a drug compound was found to be safe and effective in treating acute migraines. Caffeine may influence migraines through many possible mechanisms, mostly by adenosine receptor antagonism with further vasoconstriction and reduced CBF. Although there is a link between caffeine and migraines, a larger prospective study based on electronic diaries should be performed to assess the connection. Based on our review of the current literature, there is insufficient evidence to show that a single dose of caffeine is a migraine trigger; however, it should be emphasized that chronic caffeine overuse may lead to migraine chronification and sudden caffeine cessation may trigger migraine attacks. Migraine sufferers should be aware of the amount of caffeine they consume so that they do not exceed 200 mg daily. If they wish to continue drinking caffeinated beverages, they should keep their daily intake as consistent as possible to avoid withdrawal headache.

## Figures and Tables

**Table 1:** Overview of studies investigating the prevalence of caffeine/coffee as a trigger factor in migraineurs. TTH, tension-type headache; MWA, migraine without aura; MA, migraine with aura; EM, episodic migraine; CM, chronic migraine; TF, trigger factor.

Author (Year)	Study Design	Study Design (Method of Identifying Trigger Factors)	Study Group: Type of Headache (Number of Participants)	Study Population Age (Years)	Coffee/Caffeine Reported as a Trigger Factor (%)	Additional Information
Beh 2019 [76]	Retrospective cross-sectional	Retrospective chart review	Vestibular migraine (*n* = 131)	No data	11.5	
Tai 2018 [77]	Prospective cross-sectional	Comprehensive dietary checklist	Migraine (*n* = 319)	Migraine 37.1 ± 14.3	Migraine 25.4	Caffeine significantly associated with migraines compared to TTH
					TTH 15.1	
			TTH (*n* = 365)	TTH 46.5 ± 18.1		
			MWA (*n* = 188)			
			MA (*n* = 128)			
			CM (*n* = 91)			
Taheri 2017 [78]	Prospective observational case series	Food diary	Migraine (*n* = 65)	Range 10–15	28	87% of patients achieved complete resolution of headaches by exclusion of 1–3 triggers
			TTH (*n* = 50)	Mean 10.5		
Park 2016 [79]	Prospective cross-sectional	Smartphone headache diary application	Episodic migraine (*n* = 62)	Mean 37.7 ± 8.6	2.4	
			MWA (*n* = 60)			
			MA (*n* = 2)			
Peris 2016 [80]	Prospective cross-sectional	Detailed 90-day paper diary database from PAMINA migraine study	Migraine (*n* = 326)	No data	7.7	
Rist 2014 [81]	Cross-sectional study among participants in the Women’s Health Study	Semi-quantitative food frequency questionnaire	Non-migraine headache (*n* = 5573)	Mean 53.6	Not applicable	Patients with non-migraine headache more likely to have low intake of coffee; women who experienced migraine were less likely to have low intake of coffee compared to those with non-migraine headache
			Migraine (*n* = 7042)			
			MWA (*n* = 2972)			
			MA (*n* = 1974)			
Mollaoglu 2013 [57]	Prospective cross-sectional	Interview TF checklist	Migraine (*n* = 146)	Mean 36.32	6.3	
			MWA (*n* = 73)			
			MA (*n* = 53)			
Fraga 2013 [82]	Prospective cross-sectional	Predetermined list of trigger factors	Migraine (*n* = 100)	Range 10–20	Total 14	
					EM female 17.85	
			EM (*n* = 50)		EM male 0	
			CM (*n* = 50)		CM female 19.51	
					CM male 12.5	
Camboim Rockett 2012 [60]	Cross-sectional	Predetermined list of 22 dietary factors	Migraine (*n* = 123)	Mean 43.2 ± 13.9	**Migraine after caffeine consumption**	
			MWA (*n* = 84)			
			MA (*n* = 39)		Occasional 10–15	
					Consistent <10	
					**Caffeine withdrawal**	
					Occasional 10–15	
					Consistent 20–30	
Neut 2012 [83]	Retrospective	Predetermined list of TFs	Migraine (*n* = 102)	Mean 12	Cola drinks 8.8	
				Range 7–16		
			MWA (*n* = 71)			
			MA (*n* = 22)			
Schürks 2011 [84]	Cross-sectional	Mailed migraine-specific questionnaire	Women′s Health Study (*n* = 1675)	No data	Coffee 8.1	
					Cola drinks 5	
Yadav 2010 [85]	Prospective cross-sectional	Questionnaire	Migraine without aura (*n* = 182)	Mean 30.7	None	No subjects reported coffee or caffeine withdrawal as a trigger
				Range 14–58		
Hauge 2010 [86]	Cross-sectional	Questionnaire listing 16 trigger factors	Migraine with aura (*n* = 347)	Mean 51	Caffeine withdrawal 20–30	
Andress-Rothrock 2010 [87]	Prospective cross-sectional	Headache trigger checklist	Migraine (*n* = 200)	Mean 41.1	8	
				Range 16–75		
			EM (*n* = 56)			
			CM (*n* = 144)			
Chakravarty 2009 [88]	Prospective and retrospective cross-sectional	Migraine trigger checklist	Migraine (*n* = 200)	Range 7–15	Caffeinated drinks	
			MWA (*n* = 197)		Retrospective study 0	
			MA (*n* = 3)			
					Prospective study 0	
Fukui 2008 [89]	Prospective cross-sectional	Predetermined list of TGGs	Migraine (*n* = 200)	Mean 37.7	14.5 (12.96% females, 21.05% males)	
Wöber 2006 [90]	Cross-sectional	Two predetermined TF checklists (patients′ personal experience and theoretical knowledge)	Migraine (*n* = 71)	Range 18–65	Theoretical knowledge 25	Difference between theoretical knowledge and personal experience of coffee was statistically significant
					Personal experience 10	
			TTH (*n* = 49)	Migraine 36.8 ± 11.4		
				TTH 39.5 ± 12.7		
Takeschima 2004 [91]	Door-to-door survey	Structured questionnaires	Headache (*n* = 1628)	No data	None	Odds ratio of coffee and tea consumption significantly higher in migraineurs compared to TTH sufferers
			migraine (*n* = 342)			
			MWA (*n* = 301)			
			MA (*n* = 41)			
Bank 2000 [92]	Population-based epidemiological survey	Self-administered headache questionnaire	Migraine (*n* = 62)	Mean	None	
				Women 41		
				Men 43		
Van Den Bergh 1987 [93]	Retrospective	Unstructured recall/free self-report	Migraine (*n* = 217)	Mean 40	6.4	
